# A Case of Occlusion of the Vagina with Retention of the Catamenia

**Published:** 1870-12

**Authors:** 


A Case of Occlusion of the Vagina with Retention of the Catamenia, is reported by Dr. J. B. Mallory, in a woman who had borne five children. After weaning her last child she menstruated three months regularly, and then ceased for five months. At this time excessive pains and hemorrhage ensued, and in a short time there was passed from the vagina a fleshy, fibrous mass of the size and form of an egg. The pain then ceased, but returned in slight paroxysms at intervals for five or six months, during which time her menstrual function did not return.

Twelve months after cessation of the flow she was attacked with such severe pains in the region of the pelvis that she came to the writer. On examination per vaginum an obstruction was found that completely occluded the canal. The obstruction was diagnosed as an adhesion of the vaginal walls, which had probably taken place after the discharge of the egg-shaped mass. Back of the obstruction there was a large tumor, evidently accumulated secretion from the uterus.

A small opening was made through the septum, which was found to be an inch and more in thickness, and a tarry fluid flowed out. She continued to improve for about three months, when an inflammation occurred in the left iliac region, which passed on to suppuration; subsequently the opposite side became similarly effected, discharging freely for several weeks.

The opening through the obstructing septum becoming occluded another opening had to be made subsequently; this was made larger and after some drawbacks the patient recovered her menstrual function.—*Richmond and Louisville Medical Journal.*

